# In Vitro Comparison of Two Python‐Based Programs for the Automated Analysis of Tight‐Junction Phenotype in Brain Endothelium During Bacterial Infection

**DOI:** 10.1002/cbf.70093

**Published:** 2025-06-16

**Authors:** Henry D. Mauser, Janessa Caroza, Shane Nicole Homez, Alyssa S. Arnett, William D. Cutts, Daryl W. Lam, Justin Thornton, Walter Adams, Brandon J. Kim

**Affiliations:** ^1^ Department of Biological Sciences University of Alabama Tuscaloosa Alabama USA; ^2^ Department of Biological Sciences San Jose State University San Jose California USA; ^3^ Department of Biological Sciences Mississippi State University Starkville Mississippi USA; ^4^ Department of Biological Sciences University of Texas at Dallas Dallas Texas USA

**Keywords:** blood–brain barrier, fluorescence microscopy, image analysis, tight junction disruption, tight junctions

## Abstract

Tight junction complexes are crucial features of brain endothelial cells, as they restrict the paracellular route across the blood–brain barrier. Tight junction disruption has been observed in conjunction with numerous diseases of the CNS. In such cases, the organization or integrity of cell–cell junctions may be analyzed with a variety of automated computer programs that quantitatively assess junction images. Here, we directly compare two previously developed python‐based programs—JAnaP and IJOQ— for the semi‐ or fully automated analysis of tight junctions in human stem cell‐derived brain‐like endothelial cells. Cells were infected with *S. pneumoniae* and *S. agalactiae* to initiate junction disruption, and occludin and ZO‐1 were analyzed in mock and infected groups via JAnaP and IJOQ. JAnaP and IJOQ both yielded comparable results for the quantification of tight junction disruption in brain endothelial cells. While JAnaP rendered data at the cellular level and gave more information regarding junction phenotype, IJOQ significantly reduced user time and eliminated potential user bias. Our results suggest that JAnaP and IJOQ are both appropriate for quantifying tight junction integrity in brain endothelial cells, and both may offer distinct advantages depending on their context of use.

## Summary

- Tight junction disruption is a hallmark of blood–brain barrier dysfunction. Thus, the quantification of tight junction integrity is important for understanding the mechanisms of BBB and CNS pathologies.
- Our comparison of two automated cell–cell junction analysis programs highlights the utility of both programs as a means of studying tight junction disruption in brain endothelial cells.
- Additionally, this study demonstrates the advantages of each program and suggests contexts in which one or the other may be more appropriate or beneficial.
- By evaluating the tools available for tight junction analysis, this study will promote further investigation of blood–brain barrier disruption and CNS disease.

## 1 Introduction

The blood–brain barrier (BBB) refers to the network of specialized endothelial cells that comprise the microvasculature of the brain and separate the central nervous system (CNS) from the bloodstream [1]. These cells, known as brain endothelial cells (BECs), exhibit key specializations to maintain barrier selectivity, such as an abundance of efflux transporters and nutrient importers [2, 3], suppression of transcytosis [4], and low expression of cytokines and leukocyte adhesion molecules [5, 6]. Equally important to the function of BECs at the BBB is the presence of cell–cell junction complexes, which tightly connect adjacent cells and provide a physical obstacle to paracellular transport across the barrier [7].

BEC junction complexes are composed primarily of tight junction (TJ) proteins, adherens junction (AJ) proteins, and junction scaffold proteins [1]. TJ proteins form at the apical side of the junction complex and maintain *cis* and *trans* interactions to physically connect adjacent cells [8]. At the BBB, TJs are comprised of claudins, TJ‐associated MARVEL proteins like occludin, and junction adhesion molecules (JAMs) [7]. Expression of claudin‐5, occludin, and JAM‐A is highly enriched at the BBB [7]. AJ proteins comprise an apical junction complex below the TJ complex, and function similarly to TJ proteins; In BECs, the AJ complex is comprised primarily of vascular endothelial VE‐cadherin [7]. TJs and AJs are linked to the actin cytoskeleton via scaffold proteins, primarily the protein zonula occludens‐1 (ZO‐1). ZO‐1 binds directly to claudins, occludin, and JAMs and indirectly to VE‐cadherin via α‐ and β‐catenin, and is essential for the formation of TJs and AJs [7, 8].

Dysfunction of TJs, AJs, and scaffold proteins at the BBB is a hallmark of BBB disruption. In bacterial meningitis and meningoencephalitis, junction disruption is frequently observed [9]. Gram‐positive species, including *Streptococcus agalactiae* and *Streptococcus pneumoniae* disrupt the TJ and scaffold proteins claudin‐5, occludin, and ZO‐1 [10, 11, 12]. Disruption of claudin‐5, occludin, and ZO‐1 is likewise observed during Gram‐negative *Neisseria meningitidis* [13] and *Escherichia coli* K1 infection [14], while *E. coli* K1 also disrupts VE‐cadherin function [14]. Viral meningitis may also be associated with TJ disruption, as the loss of junctional components is observed during coxsackievirus B3, herpes simplex virus 1, and SARS‐CoV‐2 infection of brain endothelium [15, 16, 17]. In other CNS pathologies, including stroke, traumatic brain injury, and psychiatric disorders such as schizophrenia and bipolar disorder, dysfunction of the junction complex at the BBB is similarly implicated [18, 19].

Consequently, the study of junction organization and expression at the BBB has become increasingly relevant. Immunofluorescent microscopy, typically with In Vitro models, is frequently utilized to characterize junction protein phenotypes during insult to the BBB or CNS. While junction images provide important qualitative observations, quantitative evaluations of junction images are often necessary. Mean fluorescent intensity of images may be quantified as a proxy measurement for junction abundance, but this metric can be compromised by nonbiological brightness aberrations between images and fails to assess junction phenotype or organization [20, 21]. Recently, several automated computer programs have been designed for the quantitative analysis of junction phenotype [20, 21, 22, 23]. These scripts provide quantification of the continuity of junction protein networks, enabling the detection of junction architecture disruption even when fluorescent intensity is unaltered.

We utilized human stem cell‐derived brain‐like endothelial cells (iPSC‐BECs or iBECs) to compare two recently developed python‐based programs for the analysis of junction phenotype—the Junction Analyzer Program (JAnaP) [21] and the Intercellular Junction Organization Quantification (IJOQ) program [20]. JAnaP is a semi‐automated program that requires the user to manually define cell borders within an image before computer analysis [21]. After the user defines the perimeter of each cell and applies a brightness threshold to eliminate background noise, the program calculates various parameters including the percent continuity of the junction protein bordering a given cell, and perpendicular or punctate junction phenotypes [21, 24]. IJOQ is a fully automated program that assesses junction disruption under In Vitro and In Vivo conditions. Because IJOQ does not require user identification of cell borders, minimal user input is needed to generate a single, unbiased value per image as an overall reflection of junction organization. IJOQ also corrects for within‐image brightness variations that may occur when there are slight deviations in the optimal z‐plane for a signal of interest [20]. While IJOQ has been shown to quantify epithelial cell disruption, it is capable of measuring junction disruption in a wide variety of model systems where the disruption causes the junctions to become discontinuous [20, 25]. Previous reports have shown that brain endothelial cells demonstrate this form of disruption in the context of various challenges [26, 27, 28]. We therefore sought to determine if IJOQ could measure BBB disruption in the context of *S. pneumoniae* infection, as well as to compare the analytical performance of JAnaP to that of IJOQ.

Here, we infected iBECs with *S. pneumoniae* and *S. agalactiae* to disrupt cell–cell junctions. After immunostaining for occludin and ZO‐1 in mock‐infected and *S. pneumoniae‐* or *S. agalactiae*‐infected cells, immunofluorescent microscopy images were captured and analyzed via JAnaP and IJOQ. *S. agalactiae* infection produced the most notable disruption in both occludin and ZO‐1, and JAnaP and IJOQ were able to detect significant differences between mock and infected groups. *S. pneumoniae* infection produced more subtle junction disruption, particularly in occludin. JAnaP and IJOQ detected significant disruption of ZO‐1 in *S. pneumoniae*‐infected cells, but only JAnaP detected significant occludin disruption. Overall, JAnaP and IJOQ produced results consistent with each other and with visual analysis. However, JAnaP was more capable of distinguishing subtle phenotype alterations and the distribution of phenotypes within an image, whereas IJOQ required significantly less user input. This study identifies the applications and limitations of these programs for cell‐cell junction analysis in brain endothelium.

## 2 Materials and Methods

### 2.1 Brain‐Like Endothelial Cell Differentiation

iPS(IMR90)−4 cells (Wicell hPSCReg ID WISCi004‐B, RRID: CVCL_C437) were differentiated to brain‐like endothelial cells (iBECs) as previously described [29, 30]. Briefly, iPSCs were maintained in Stemflex Basal Medium (Thermo cat# A3349401). Differentiation to brain‐like endothelial cells was initiated by maintaining cells in Unconditioned Media (DMEM/F12 (Thermo cat# 11320033), KOSR (Thermo cat# 10828028), NEAA (Thermo cat# 11140050), GlutaMAX (Thermo cat# 35050061, β‐mercaptoethanol) for 6 days. Endothelial cells were selectively expanded for 1 day in endothelial cell media with bFGF and retinoic acid (EC+/+) (hESFM (Thermo cat# 11111044), B27 (Thermo cat# 17504044), bFGF (PeproTech cat# 100‐18B‐50UG), retinoic acid) and then maintained in endothelial cell media without bFGF or retinoic acid (EC−/−). Then, iBECs were purified by seeding onto Collagen IV‐ and fibronectin‐coated 48‐well plates (VWR cat# 10062‐898). Two days after purification, iBECs reach maximum transendothelial electrical resistance values around 3000 Ω×cm^2^ [30, 31] and are ready for experiments.

### 2.2 Bacterial Strains and Culture

Bacterial strains used were *Streptococcus agalactiae* COH‐1 and *Streptococcus pneumoniae* TIGR4. *S. agalactiae* was grown in Todd Hewitt broth (THB) and *S. pneumoniae* was grown in THY broth (Todd Hewitt broth + 0.5% yeast extract). Before infection, *S. agalactiae* was grown in 10 mL of THB for 16–18 h, then 150–250 μL was diluted into 4 mL of fresh THB and grown to an optical density at 600 nm (OD_600_) of 0.4–0.6. Before experiments, *S. pneumoniae* was grown to an OD_600_ of 0.4 and frozen at −80°C in 15% glycerol in 1 mL aliquots. Per experiment, a 1 mL aliquot was thawed and diluted in 4 mL of fresh THY and grown to an OD_600_ of 0.4–0.6 before use.

### 2.3 Infection of IBECs

For plates infected with *S. agalactiae*, media was replaced with 200 μL of fresh media up to 2 h before infection. For both *S. agalactiae* and *S. pneumoniae*, cultures at an OD_600_ of 0.4–0.6 were pelleted at 3220*g* for 5 min and resuspended in 250 μL of phosphate buffered saline (PBS). The bacteria were then normalized in PBS to an OD_600_ of 0.4 for *S. agalactiae* or 0.2 for *S. pneumoniae*, equivalent to 1×10^8^ bacteria/mL. For *S. agalactiae*, this PBS was then diluted 1:10 into EC−/− media, and 50 μL was used to infect each well to achieve a multiplicity of infection (MOI) of 10. Plates infected with *S. agalactiae* were centrifuged at 200*g* for 5 min and incubated at 37°C + 5% CO_2_ for 5 h. For *S. pneumoniae*, the normalized PBS was diluted 1:25 into EC−/−, and 125 μL of media was used to infect cells at an MOI of 10, which were then incubated at 37°C + 5% CO_2_ for 3.5 h.

### 2.4 Immunostaining and Microscopy

**Figure 1:**
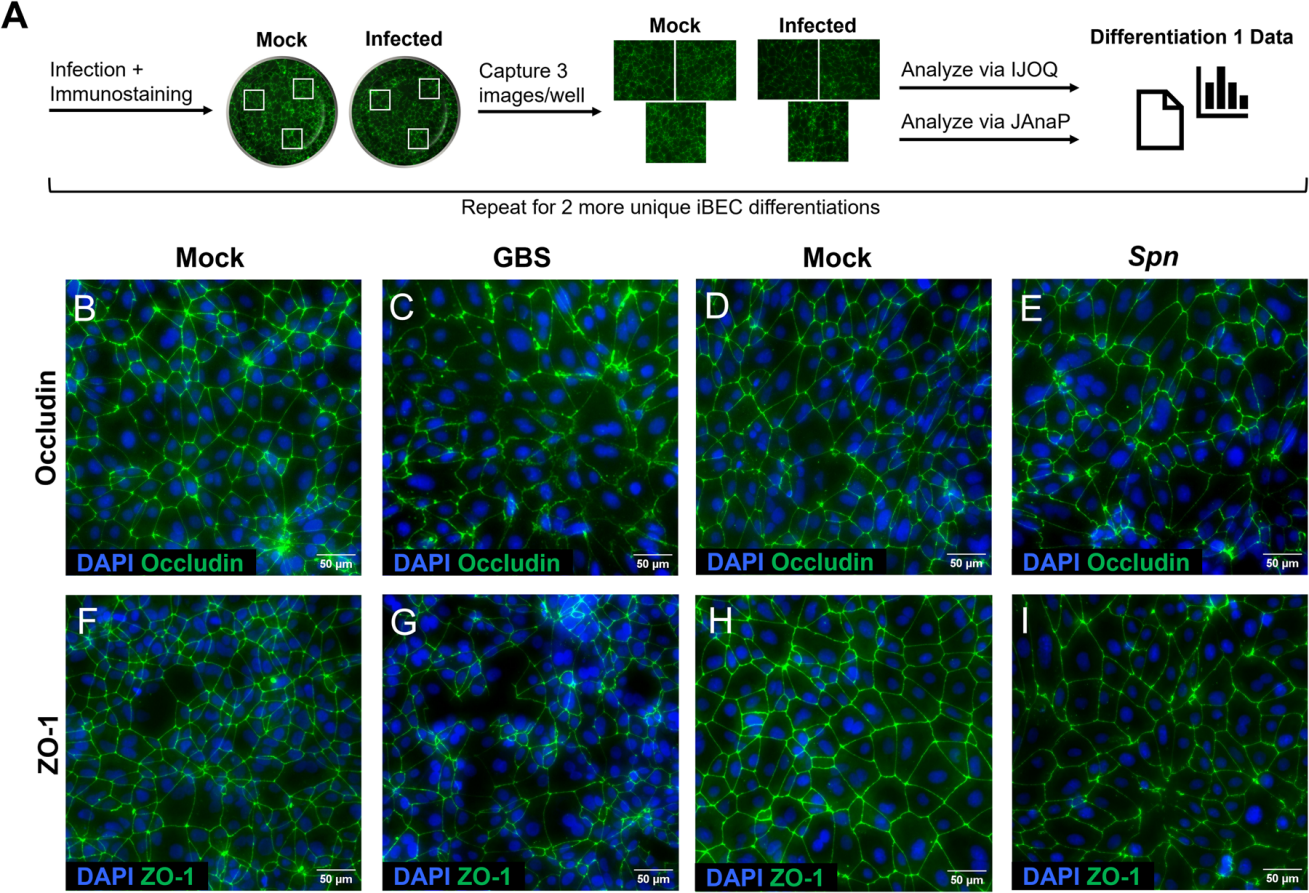
Occludin and ZO‐1 staining in mock vs. *S. agalactiae*‐ (GBS) or *S. pneumoniae*‐ (Spn) infected iBECs. (A) Flow chart for collection of images from mock vs. infected conditions for each iBEC differentiation. (B–I) Representative immunofluorescent images of iBECs infected with *S. agalactiae* COH‐1 for 5 h at an MOI of 10 (B and C, F and G) or *S. pneumoniae* TIGR4 for 3.5 h at an MOI of 10 (D and E, H and I). Cells were immunostained and imaged to detect occludin (B–E) and ZO‐1 (F–I), displayed in green. Cell nuclei are visualized by DAPI, displayed in blue. Images were overlayed in ImageJ and cropped for closer visualization. Scale bars and legends are shown.

After infection, cells were fixed with ice cold methanol for 15 min, washed 3× with PBS, and blocked with 10% fetal bovine serum (FBS) in PBS for at least 1 h. Cells were incubated with the primary antibody overnight at 4°C. After washing 3× with PBS, secondary antibody was applied for 1 h. Cells were washed, DAPI solution (VWR cat# PK‐CA70740043) was applied for 15 min, and cells were washed once more before imaging. Three images were captured within each well of the 48‐well plate on an Eclipse Ti2‐E inverted microscope (Nikon). All images were captured with identical settings relative to their controls. ImageJ 2.0 (RRID: SCR_003070) was used to crop images to 1024 × 1024 pixels for analysis and to apply scale bars for image presentation. Our workflow for image collection and analysis is depicted in Figure 1A.

### 2.5 Antibodies and Dilutions

Occludin monoclonal, 1:200 (Thermo cat# 33‐1500, RRID: AB_2533101); ZO‐1 monoclonal, 1:100 (Thermo cat# 33‐9100, RRID: AB_2533147); Goat anti‐mouse Alexa Fluor 488 secondary, 1:200 (Thermo cat# A11001, RRID: AB_2534069).

### 2.6 Python‐Based Program Analyses

The IJOQ (RRID: SCR_026026) and JAnaP (RRID: SCR_026027) programs were used as previously described [20, 21]. Both scripts are publicly accessible and available for download at https://github.com/DevonsMo/IJOQ/releases (IJOQ) and https://github.com/StrokaLab/JAnaP (JAnaP). Briefly, to analyze images via IJOQ—the program was calibrated using the advanced settings with mock‐infected images to generate the calibration settings. Users examined the calibration output images and adjusted the settings to ensure adequate thresholding. IJOQ analysis was performed on all images, with two users per condition. Because determining IJOQ settings requires limited user input, user bias is minimal. No changes to the IJOQ program code were made to perform the image analysis. For JAnaP, all nonadjacent cells per image were manually defined via waypointing, typically yielding 30–50 cells per image. A threshold value was determined based on a representative cell from the mock condition and then used to filter out background fluorescence in each image. To limit potential user bias, each biological replicate within a biological triplicate was analyzed by a different user, for a total of three users per condition. The JAnaP “Fast Class” feature was utilized to analyze all waypointed images.

### 2.7 Statistics and Data Presentation

Nine images were analyzed per condition, comprising of technical triplicates from three independent iBEC differentiations. For all IJOQ analyses, *n* = 9, as IJOQ renders a single value per image analyzed. An unpaired Student's *t*‐test was used for all IJOQ comparisons and to compare user times between IJOQ and JAnaP. For all JAnaP analyses, *n* is variable, as JAnaP renders a single value for each cell within an image. For JAnaP data, the non‐parametric Mann–Whitney–Wilcoxon test was used. Significance was determined as *p* < 0.05. Statistics were performed and graphs were made in GraphPad Prism 10.30.0 (RRID: SCR_002798).

## 3 Results

### 3.1 *S. agalactiae* and *S. pneumoniae* Disrupt Occludin and ZO‐1 in Brain Endothelium

To disrupt TJs in iBECs, we infected monolayers with *S. agalactiae* or *S. pneumoniae*. *S. agalactiae* is the leading cause of neonatal bacterial meningitis, and *S. pneumoniae* is the leading cause of bacterial meningitis in adults [32, 33]; both species disrupt TJs in brain endothelium. *S. agalactiae* disrupts the TJ protein occludin and the scaffold protein ZO‐1 in brain endothelium, among other junction components [10, 12]. Likewise, *S. pneumoniae* disrupts ZO‐1 in BECs and recent work demonstrates that pneumococcal extracellular vesicles induce the autophagic degradation of occludin in alveolar epithelium [11, 34]. Thus, after infecting iBECs with *S. agalactiae* or *S. pneumoniae*, cells were stained with occludin or ZO‐1 antibodies and visualized by immunofluorescent microscopy (Figure 1B–I). Both occludin and ZO‐1 disruption were readily observable in *S. agalactiae*‐infected cells, compared with the mock, as indicated by lower intensity and intermittent staining along cell boundaries (Figure 1B,C,F,G). ZO‐1 disruption was likewise apparent in *S. pneumoniae*‐infected cells, although ZO‐1 appeared more continuous here than in the *S. agalactiae*‐infected equivalent (Figure 1D,E). Conversely, differences in occludin between mock and *S. pneumoniae*‐infected cells were only subtly apparent (Figure 1H,I). Even where junction disruption was apparent, fluorescent intensity between mock and infected often appeared similar (Figure 1B,C, H,I), which further motivated the utilization of computer‐automated junction phenotyping.

### 3.2 IJOQ and JAnaP Analyses Suggest Junction Disruption in *S. pneumoniae*‐ and *S. Agalactiae*‐ Infected Groups

**Figure 2:**
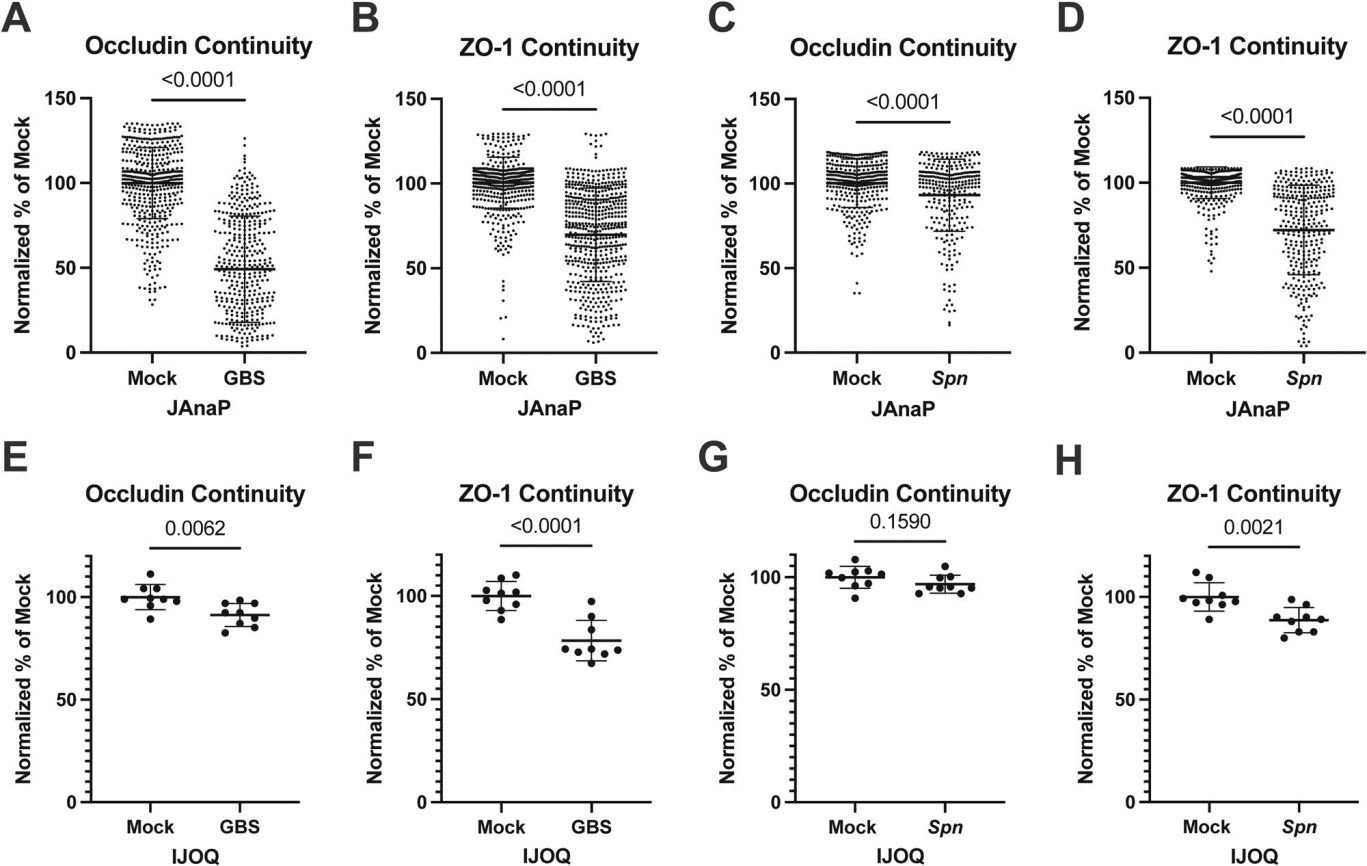
IJOQ and JAnaP analyses of occludin and ZO‐1 in mock vs. *S. agalactiae*‐ (GBS) or *S. pneumoniae*‐ (*Spn)* infected iBECs. (A–D) Percent continuity as determined by JAnaP in occludin (A, C) or ZO‐1 (B, D) in mock vs. GBS (A, B) or mock vs. *Spn*‐ (C, D) infected cells. (E, H) IJOQ values in occludin (E, G) or ZO‐1 (F, H) in mock vs. GBS (E, F) or mock vs. *Spn*‐ (G‐H) infected cells. Nine images were analyzed per condition, comprised of technical triplicates from three independent iBEC differentiations. The *n* values for JAnaP are as follows: occludin mock vs. GBS, *n* = 556 and *n* = 396, respectively; ZO‐1 mock vs. GBS, *n* = 557 and *n* = 562, respectively; occludin mock vs. *Spn*, *n* = 366 and *n* = 289, respectively; ZO‐1 mock vs. *Spn*, *n* = 438 and *n* = 318, respectively. For IJOQ analyses, *n* = 9. Error bars represent SD. Mann–Whitney–Wilcoxon test was utilized to determine significance between mock and infected groups for JAnaP (A–D), and Student's *t*‐test for IJOQ (E–H). *p* values are shown. Significance is defined as *p* < 0.05.

To quantitatively assess TJ disruption, the immunofluorescent images were analyzed via JAnaP and IJOQ (see Section 2). In JAnaP, the user manually defines the cell border by “waypointing” and the program determines the percentage of that border that is occupied by pixels that exceed the background threshold [21]. Consequently, a percent continuity measurement is generated for each cell that is waypointed, allowing JAnaP to capture the distribution of junction phenotypes within an image. For each of the four conditions, JAnaP continuity measurements indicated that *S. pneumoniae* and *S. agalactiae* induced a significant loss of occludin and ZO‐1 continuity (Figure 2A–D). IJOQ, in contrast, analyzes junction integrity by overlaying a grid on threshold‐corrected images and recording the intersection between grid lines and junctions [20]. Thus, IJOQ generates a single value per image analyzed, providing less information than JAnaP. Nevertheless, IJOQ analysis produced comparable results to JAnaP, detecting a significant loss of occludin in the *S. agalactiae*‐infected group and of ZO‐1 in both conditions (Figure 2E–H).

### 3.3 JAnaP Offers More Information While IJOQ Limits Operator Time and Bias

In addition to percent continuity measurements, JAnaP calculates other parameters, namely the percentages of a cell's junctions that are punctate, perpendicular, and discontinuous [21]. A junction becomes punctate when *trans* interactions between junctions on two cells become disrupted, and the junction complex begins to separate, forming puncta. A perpendicular junction phenotype indicates the ectopic formation of a junction perpendicular to the border of the cell. By assessing these disruption‐associated phenotypes, JAnaP detects junction disruption beyond the simple junction loss or breakage [21, 24]. JAnaP analysis of infected cells suggested that the incidence of perpendicular and punctate junctions increased significantly in *S. agalactiae*‐ and *S. pneumoniae*‐infected cells (Figure S1A–D). Consistent with the findings for junction continuity, the incidence of perpendicular and punctate occludin appears to be less significantly altered by *S. pneumoniae* infection than by *S. agalactiae* infection, or than ZO‐1 is in either condition (Figure S1A–D).

**Figure 3:**
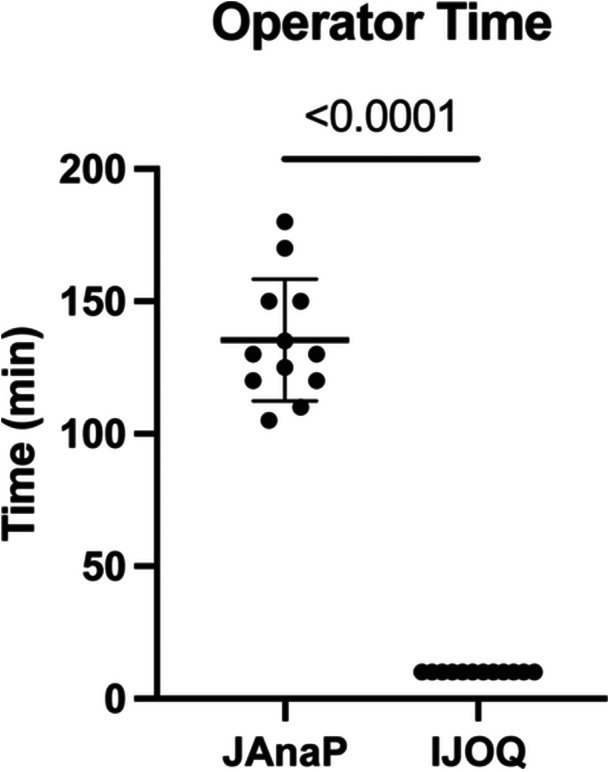
JAnaP vs. IJOQ user time per image set. One image set consisted of three mock and three infected images for either occludin or ZO‐1. User time is defined as the time from initializing the program to the generation of results. Data are displayed as the mean of all replicates, comprised of technical triplicates from each of the four conditions analyzed in Figure 2. Error bars represent SD. Student's *t*‐test was utilized to determine significance between JAnaP and IJOQ. *p* value is shown. Significance is defined as *p* < 0.05.

Because IJOQ is fully automated, it suggests distinct benefits such as limiting operator time and reducing the potential for operator bias. To determine the true time discrepancy between operating IJOQ and JAnaP, user time was recorded from the initialization of either program to the generation of the results for each of the three biological replicates within all four conditions. As each of these 12 image sets was analyzed separately by JAnaP, individual user times were recorded for each image set. While JAnaP required an average of 130 min per image set, IJOQ averaged a mere 10 min per image set, reflecting a 13‐fold decrease in operator time required (Figure 3).

## 4 Discussion

Analysis of cell–cell junction disruption in BBB models is an important aspect of quantifying CNS pathologies, including bacterial and viral meningitis/meningoencephalitis, ischemic stroke, and psychiatric disorders [9, 35]. The growing importance of understanding cell–cell junction disruption in these diseases has spurred the development of computer programs to quantify junction disruption observed by immunofluorescent microscopy. These junction analysis programs improve upon previous methods—rather than using fluorescent intensity measurements, they incorporate computer automation to determine junction continuity and other phenotypes [20, 21, 22, 23].

Of these tools, Tight Junction Organization Ratio (TiJOR) and IJOQ are the most similar, as they require insignificant user input and generate a single data point per image analyzed. Functionally, they rely on the intersection of junction pixels with an artificial grid or shape overlayed on the image. Additionally, both tools were validated In Vitro using human lung epithelial cell lines and In Vivo with murine models [20, 23]. IJOQ improves upon TiJOR, however, by applying a brightness normalization algorithm to each image before processing, which corrects for brightness variations due to z‐plane deviations. Moreover, in a direct comparison between IJOQ and TiJOR, IJOQ was found to detect disruption in AJs and TJs where TiJOR did not, or degrees of disruption which TiJOR could not distinguish [20]. Thus, in addition to the greater degree of automation that IJOQ provides over TiJOR, which limits bias and improves repeatability, IJOQ is the more discriminating of the two tools.

Junction Mapper and JAnaP are similar in that they require considerable user input but generate a variety of phenotypic information for individual cells within an image. Junction Mapper was validated using keratinocytes, cardiomyocytes, and human umbilical vein endothelial cells [22]. The program uses the manual and automatic identification of junction foci and cell–cell contacts to quantify junction length, intensity, and area [22]. In contrast, JAnaP was designed to analyze BBB junction phenotype and was validated in human primary BECs [21]. Consequently, JAnaP is frequently used to investigate BBB disruption [24, 36, 37, 38]. JAnaP utilizes user‐defined cell borders to calculate primary parameters such as perimeter, area, and junction coverage, and secondary parameters including the percent of continuous, perpendicular, punctate, and discontinuous junctions [21].

While we chose to quantify these basic parameters with the JAnaP “Fast Class” analysis function (Figures S1, 2), the JAnaP “Junction Class” function is able to generate additional data to express junction phenotype in terms of more detailed parameters. These parameters include start and end index, length, aspect ratio, tip‐to‐tip distance, and others that quantify individual junction segments per cell. Segments may be as small as a few pixels, giving the user detailed information regarding all regions of the cell border. These data are then used to classify junction segments as punctate, perpendicular, or continuous. Thus, while the analysis we utilized expresses continuity parameters as a percentage of each cell, JAnaP can provide a far more detailed report of cell junction status. While “Junction Class” analysis requires significantly more program time, it does not require additional user time. Additionally, JAnaP can process multiple channels of the same image simultaneously if the user wishes to stain for multiple proteins with different secondary fluorophores. While this feature is not utilized in the present study, it is another valuable capability of the program.

Previously, JAnaP has assessed occludin and ZO‐1 disruption in brain endothelium [21, 36], whereas IJOQ has assessed junction disruption in the lung and intestinal epithelium [20, 25, 39, 40]. We investigated whether IJOQ could detect BBB disruption, and how it compared to JAnaP, a well‐utilized tool in the BBB field. Notably, IJOQ values and JAnaP % continuity measurements were comparable for occludin and ZO‐1 in either the *S. agalactiae*‐ or *S. pneumoniae*‐infected conditions (Figure 2). In the one instance where JAnaP found a significant difference between mock and infected when IJOQ did not (Occludin, Mock vs. *Spn*) (Figure 2C,G), IJOQ still reflected a slight decrease in the mean of the infected group versus the mock. While JAnaP represents a similarly small difference between mock and infected here, the abundance of cells quantified per condition allows JAnaP to detect this slight difference. As the results of IJOQ generally agree with those derived from JAnaP, this study shows that IJOQ is capable of accurately measuring tight junction disruption in the BBB. Notably, this is the first report of *S. pneumoniae*‐dependent disruption of occludin in brain endothelium. Given the novel and nuanced nature of this defect as well as the different results between IJOQ and JAnaP, further study of this phenotype is warranted. Ultimately, while JAnaP may be more beneficial for analyzing subtle junction disruption phenotypes, IJOQ nevertheless was able to reliably detect most instances of iBEC junction disruption.

Because IJOQ was designed to generate a single metric—intercellular junction organization—it does not measure the additional phenotypes that are assessed by JAnaP. However, this study does demonstrate multiple key benefits that IJOQ offers compared with JAnaP and other semi‐automated analysis programs. One clear advantage is the time that IJOQ saves. Compared with JAnaP, IJOQ spared around 2 h of operator time per image set or 24 h total for all the image sets (Figure 3). Furthermore, IJOQ's minimal user input naturally eliminates many sources of potential bias that might impact image analysis. To control for user bias in our JAnaP analyses, each of the three biological replicates was analyzed by a different user. However, IJOQ does not necessitate multiple users, and it is rapid enough for a single user to reasonably analyze hundreds of images. Additionally, JAnaP may be less than ideal for analyzing severe disruption as the program requires manual cell tracing. If junctions are so disrupted that the user cannot infer where cell borders are, then JAnaP is unusable. Thus, in scenarios of severe disruption, IJOQ may be more applicable, as it does not require cell border identification.

JAnaP and IJOQ offer distinct advantages for unique contexts, but they may also be synergistic. As IJOQ is high throughput and eliminates user bias, it might be utilized as a screening tool to identify conditions that merit further analysis. Images that pass IJOQ screening could then be analyzed via JAnaP to generate more specific phenotypic information. Overall, our results support the use of IJOQ and JAnaP for analyzing junction disruption in brain endothelium and motivate the continued study of these and other programs to optimize junction analysis. The use of these tools in appropriate contexts will enable junctional protein image quantification at the BBB and provide a more comprehensive understanding of how unique disease states impact brain endothelial integrity.

## Conflicts of Interest

The authors declare no conflicts of interest.

## Supporting information

Figure S1. JAnaP raw data for % continuous, % punctate, % perpendicular, and % discontinuous junctions. (A–B) Occludin and ZO‐1 in mock vs. *S. agalactiae*‐(GBS) infected cells. (C–D) Occludin and ZO‐1 in mock vs. *S. pneumoniae*‐(*Spn)* infected cells. Error bars represent SD. Mann–Whitney–Wilcoxon test was utilized to determine significance between mock and infected groups. *p* values are shown. Significance is defined as p < 0.05.

## Data Availability

The authors confirm that all raw image and image analysis data supporting the findings of this study are available at the https://doi.org/10.6084/m9.figshare.28049660.v1. All other data are present in the manuscript or its supplementary materials.
