# Supplementary figures and images for: In Vitro Comparison of Two Python‐Based Programs for the Automated Analysis of Tight‐Junction Phenotype in Brain Endothelium During Bacterial Infection
Source: Cell Biochem Funct. 2025 Jun 16;43(6):e70093. doi: 10.1002/cbf.70093 (PMC12169080; doi:10.1002/cbf.70093)

**A**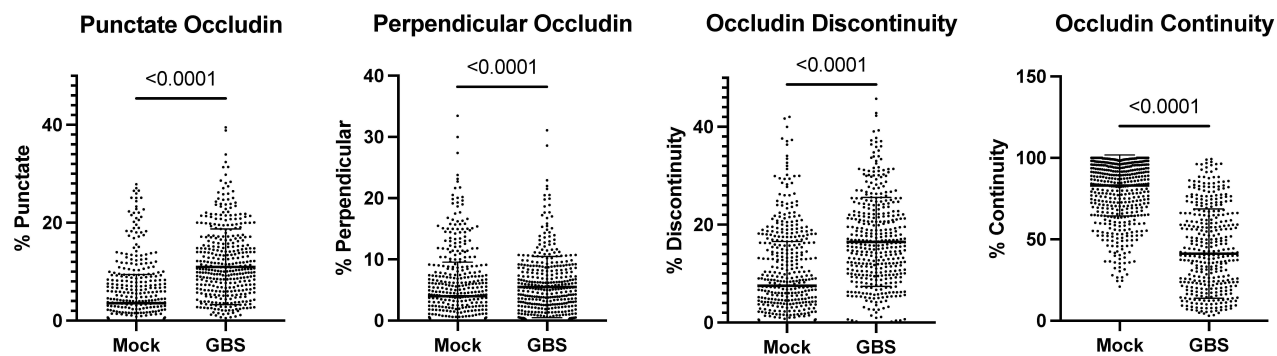**B**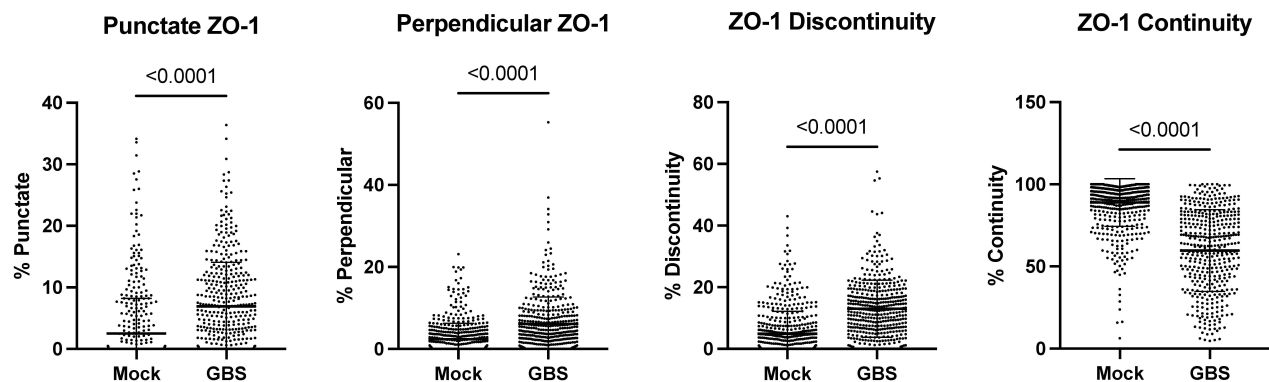**C**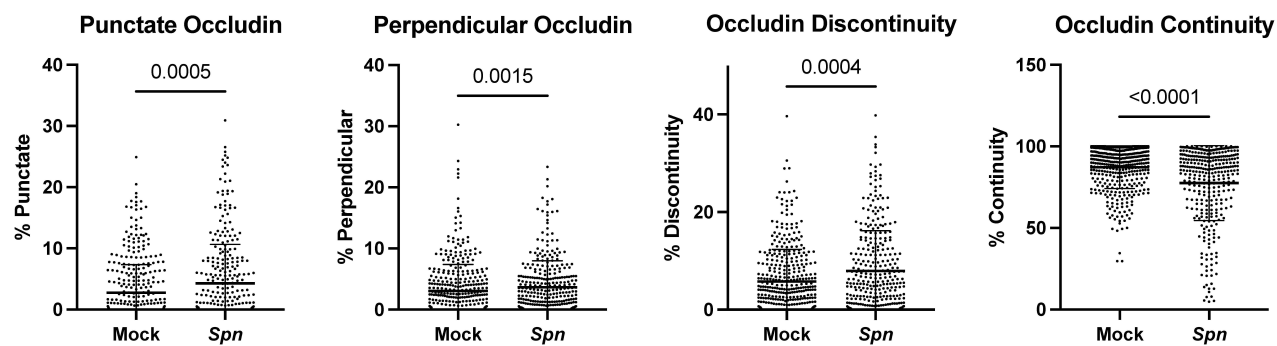**D**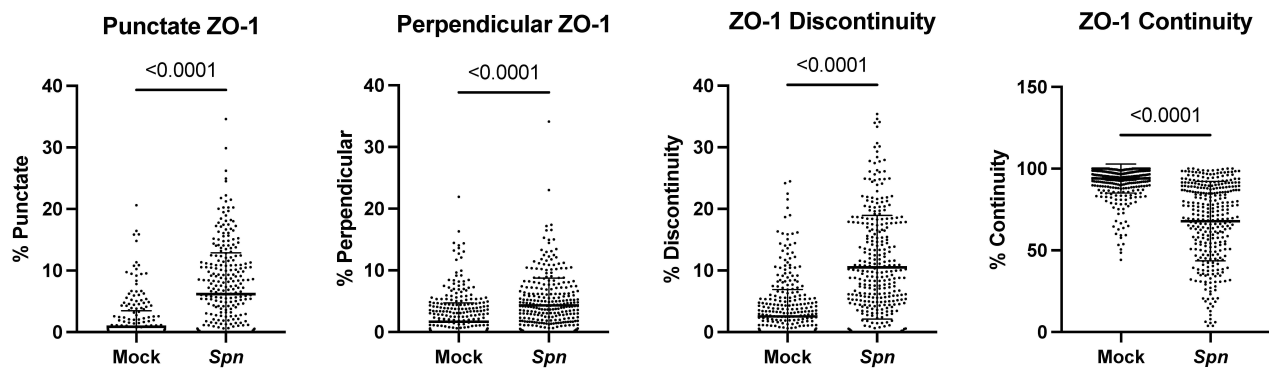

Supplement: Supplementary file 1 — Figure S1. JAnaP raw data for % continuous, % punctate, % perpendicular, and % discontinuous junctions. (A–B) Occludin and ZO‐1 in mock vs. S. agalactiae‐(GBS) infected cells. (C–D) Occludin and ZO‐1 in mock vs. S. pneumoniae‐(Spn) infected cells. Error bars represent SD. Mann–Whitney–Wilcoxon test was utilized to determine significance between mock and infected groups. p values are shown. Significance is defined as p < 0.05. [file CBF-43-e70093-s001.pdf]
